# Designing the next generation of implementation research training for learners in low- and middle-income countries

**DOI:** 10.1186/s12992-021-00714-3

**Published:** 2021-06-21

**Authors:** Michael J. Penkunas, Shiau Yun Chong, Emma L. M. Rhule, Evangelia Berdou, Pascale Allotey

**Affiliations:** 1 United Nations University International Institute for Global Health (UNU-IIGH), UKM Medical Centre Jalan Yaacob Latif, Bandar Tun Razak, Cheras, 56000 Kuala Lumpur, Malaysia; 2 Data Yarns Research, Brighton, England

**Keywords:** Implementation research, Training, Research capacity strengthening

## Abstract

Efficacious health interventions tested through controlled trials often fail to show desired impacts when implemented at scale. These challenges can be particularly pervasive in low- and middle-income settings where health systems often lack the capacity and mechanisms required for high-quality research and evidence translation. Implementation research is a powerful tool for identifying and addressing the bottlenecks impeding the success of proven health interventions. Implementation research training initiatives, although growing in number, remain out of reach for many investigators in low- and middle-income settings, who possess the knowledge required to contextualize challenges and potential solutions in light of interacting community- and system-level features. We propose a realigned implementation research training model that centers on team-based learning, tailored didactic opportunities, learning-by-doing, and mentorship.

## Background

An ever-growing number of efficacious interventions for infectious and non-communicable diseases are available to health systems charged with scaling lifesaving programs. Yet, scientifically tested solutions often fail to demonstrate desired impacts when implemented through national disease control programs or population health initiatives [1]. The need to address implementation bottlenecks is often greatest in low- and middle-income countries (LMICs), where health systems typically lack established mechanisms to effectively collect and use locally generated information [2]. Building capacity to identify implementation challenges and enact strategies for overcoming bottlenecks is increasingly vital as countries strive for universal health coverage. Researchers and practitioners working in LMICs are in the best position to produce locally generated evidence to ensure interventions are implemented optimally, remain relevant, and achieve sustainability.

Implementation research (IR) is the systematic approach to improving access to efficacious health interventions, strategies, and policies through understanding and addressing barriers to effective implementation and scaling [3]. IR provides the evidence to develop strategies to improve access and uptake of health interventions by the populations in need [3], and can play a critical role in improving the delivery of disease control interventions. The increasing interest in IR and growing demand for IR training has inspired a range of training programs, from mini-courses, workshops, and bootcamps, to fully accredited postgraduate courses [4]. The majority of these offerings are provided by institutions in North America and Europe, with most courses out of financial and practical reach for aspiring IR investigators in LMICs. Additionally, the needs of investigators in LMICs can easily be overlooked or undervalued when these trainings are conceptualized and developed in high-income settings. There are a small number of notable academic courses that actively recruit and subsidize students from LMICs [5], but the demand for these specialized programs far outstrips the number of places available.

The expanding importance of online learning has spurred a suite of open access, web-based IR training tools, with some materials specifically targeting learners in LMICs. There are freely accessible massive open online courses that provide learners with a thorough understanding of IR and the essential skills necessary to carry out an IR project [6]. Supplementing online courses, a library of toolkits and guides are available for investigators in LMICs wishing to gain skills in IR. Notably, a growing number of these materials are available in languages other than English, including French, Spanish, Arabic, Chinese, and Russian [7]. Yet, challenges in accessing high-quality training persist, and there remains a need to develop a holistic training program designed to meet the needs of IR investigators in LMICs.

Acknowledging the promise that IR holds, combined with the need for additional training opportunities, the United Nations University International Institute for Global Health mapped current training opportunities and identified points of leverage to enhance IR offerings. We carried out a review of the academic and grey literature, interviewed developers of IR courses for learners in LMICs (eight of ten interview participants from LMICs), and held a virtual dialogue series with champions of IR trainings (40 of 66 dialogue participants from LMICs). The process engaged IR training experts representing 18 countries globally. The data from these combined sources were synthesized under four emergent foundational principles: team-based learning, tailored didactic opportunities, learning-by-doing, and mentorship. Based on these principles, we propose a revised IR training model.

## Team-based learning

In practice, IR is best conducted by a trans-disciplinary team composed of researchers (i.e., health service researchers, economists, anthropologists, among others), health service implementors, and policy-makers [8]. This mix of epistemic and practical skillsets ensures the IR project is scientifically rigorous, considers the needs of the program under study, and possesses the political buy-in to translate findings into actionable changes. Yet, IR courses typically focus on training a single individual, most often a researcher, who may not have the subject matter expertise to fully understand implementation challenges nor the political power to enact large-scale programmatic change. The new model redefines the unit of training as the IR team itself. The aim is to recruit and train a trans-disciplinary group of individuals who form a fully functional IR team capable of planning and executing an IR project. The team could come from a single institution, such as a Ministry of Health, or could draw from various institutions who collaborate to solve health challenges, such as universities, national disease programs, and non-governmental organizations. The inclusion of policy-makers within the team helps ensure the study findings will be integrated into the decision-making process while simultaneously elevating the profile of IR and promoting the adoption of IR for evidence generation among policy audiences. Championing research findings into action within complex systems and securing funding for sustaining revised implementation strategies are among the essential functions of policy-makers within the IR team.

## Tailored didactic opportunities

Two opportunities exist to tailor didactic materials to meet the needs of learners in LMICs. First, regionally-specific case examples and IR project walkthroughs are in high demand. A library of case studies for challenges and diseases endemic to South America, Asia, and the Middle East will complement the large number of IR examples currently available for projects carried out in sub-Saharan Africa. The added value for learners is clear, as they will be able to better identify with examples that reflect challenges they encounter themselves. Second, considering the composition of the IR team, opportunities exist to develop content that is specific to the roles individuals on the team play. For example, materials targeting policy-makers could focus less on study design and more on how to apply IR findings when making decisions around programmatic changes [9]. Content for health service implementors could focus on strategies for community engagement as well as identifying bottlenecks associated with implementation outcomes (e.g., acceptability, fidelity, penetration). Researchers currently have the largest number of didactic resources available to them, but there is a need to supplement the current offerings. Some researchers may have many years of experience in health or social science but are new to IR. These investigators could benefit from materials that focus on the nuances associated with IR rather than basic research methods. The potential to tailor IR activities to address health inequity is largely untapped, and the next generation of training should enable research teams to harness the power of IR to identify and address factors driving inequity [10]. In support of this, efforts are underway to integrate gender and intersectionality concepts into IR training [11]. Additional content on results translation, team building, and community engagement are also in demand. Fundamentally, the core concepts of IR should be understood by all members of the IR team [12], but the learning goals for each team member could reflect the role in which they play. Creating tailored didactic content leverages the expertise of each team member and can help maintain interest by excluding training requirements that are not applicable to each individuals’ role on the IR team.

## Learning-by-doing

Incorporating a pool of funding to complete an IR project into the training program allows the IR team to rapidly apply the skills developed during the didactic training component. The integration of research funding enables IR teams to immediately apply their training and coalesce around a project tackling a local implementation challenge. In practice, IR teams will likely have identified a topic to study before enrolling in the training program. The team will apply their newly acquired skills to design a research protocol, data collection tools, and results translation plans for an applied project, extending the didactic courses into a hands-on training component. Several applied research grants are available for health researchers in LMICs [13], and partnering with funders could ensure financing is available for the practice-based component of this team-based model.

## Mentorship

Underpinning the didactic and learning-by-doing components is a strong emphasis on mentorship for the IR team. The need for mentorship for newly trained IR investigators emerged as a repeated theme in the literature review and dialogues with IR training experts and past students. The ever-growing number of institutes developing IR training courses, some of which are hosted in LMICs, possess a wealth of knowledge and could serve as mentorship pools as the IR teams carryout their initial research project. The mentors, matched with IR teams sharing similar research interests, can advise on experimental design, data collection techniques, and results translation approaches while simultaneously connecting the IR team to the larger network of IR professionals. The expertise housed within these centers and universities can also be leveraged to lead aspects of the didactic training component in addition to filling the demand for IR mentorship. Importantly, this relationship can serve as a two-way conduit of both individuals and knowledge between the new training program and the mentorship institutions: Individuals completing the team-based training program can connect with mentoring universities for continued education and research opportunities, just as graduates from these universities could take part in this newly designed team-based training program.

## Conclusions

Information collected through our IR training mapping and consultations with IR champions and past students identified gaps in the current IR training landscape for LMIC learners. The proposed training model we developed to address these gaps is not a grand overhaul of what currently exists - instead, our model represents an alignment of the currently available courses, resources, and centers to expand the opportunities for learners in LMICs to become skilled IR scientists. The advancement of team-based learning, creation of regionally- and learner-specific IR training content, and emphasis on mentorship are the most substantive innovations represented in this model. Acknowledging that high-quality trainings exist and are continuously growing in number, we attempt to connect resources meaningfully to provide learners in LMICs with a training package that will instill the skills needed to identify and overcome bottlenecks to best implement lifesaving interventions locally. Importantly, this model attempts to both increase the number of trained IR investigators in LMICs and expand access to high-quality trainings to program implementors and policy-makers, which has the potential to enhance the relevance of IR conducted locally and improve the uptake of research findings [14]. Moving forward, we are developing a detailed learning, recruitment, and outcomes framework for this program, with an anticipated launch in 2021.

## Data Availability

Not applicable.
